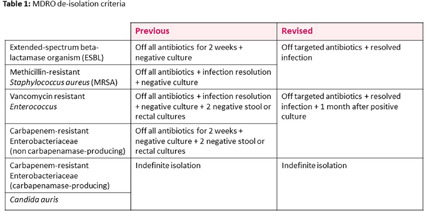# Impact of Shorter Contact Isolation Duration on Healthcare Associated Multidrug-Resistant Organisms in a Pediatric Medical Center

**DOI:** 10.1017/ash.2025.205

**Published:** 2025-09-24

**Authors:** Ayelet Rosenthal, Nabgha Farhat, Sneha Krishna, Joseph Fishbein, Amy Valencia, Alison Prati, Julianne Burns, Roshni Mathew

**Affiliations:** 1 Lurie Children’s Hospital of Chicago, Northwestern University; 2 Lurie Children’s Hospital; 3 Stanford Medicine Children’s Health; 4 Stanford Medicine Children’s Health; 5 Stanford University School of Medicine; 6 Stanford University

## Abstract

**Background:** Patients with multidrug-resistant organisms (MDROs) often require prolonged contact isolation, negatively impacting patient care and resource utilization. De-isolation criteria for MDROs vary across pediatric hospitals, typically based on organism type and achieving negative cultures. This study assessed the impact of revised MDRO de-isolation criteria allowing shorter contact isolation (Table 1) on healthcare-associated (HA) MDRO incidence rates in a freestanding academic pediatric medical center. **Methods:** We measured HA-MDRO incidence (MDROs listed in table 1, identified on or after hospital day 3) per 1000 patient days during two periods: (1) Pre-intervention (January 2019 – February 2022), prior to revised de-isolation criteria, and (2) Post-intervention (March 2022 – July 2024). Negative binomial regression was used to compare the level and trend of HA-MDRO incidence rates between the periods. **Results:** The incidence rates of all HA-MDROs, extended-spectrum beta-lactamase (ESBL)-producing organisms and methicillin-resistant Staphylococcus aureus (MRSA) are shown in Figure 1. No significant difference was observed in the level (p=0.38, 0.37, 0.9) or trend (p=0.67, 0.82, 0.76) of HA-MDRO, ESBL, or MRSA incidence rates between the periods. Estimating a daily cost of about $43 for personal protective equipment only, a minimum reduction of two weeks of contact isolation translates to approximately $602 cost reduction per patient. **Conclusion:** Shortening the duration of contact isolation for MDROs did not increase HA-MDRO incidence rates in our children’s hospital and may offer cost savings. Carefully designed MDRO policies can enhance patient care without compromising infection prevention goals.

**Figure:**
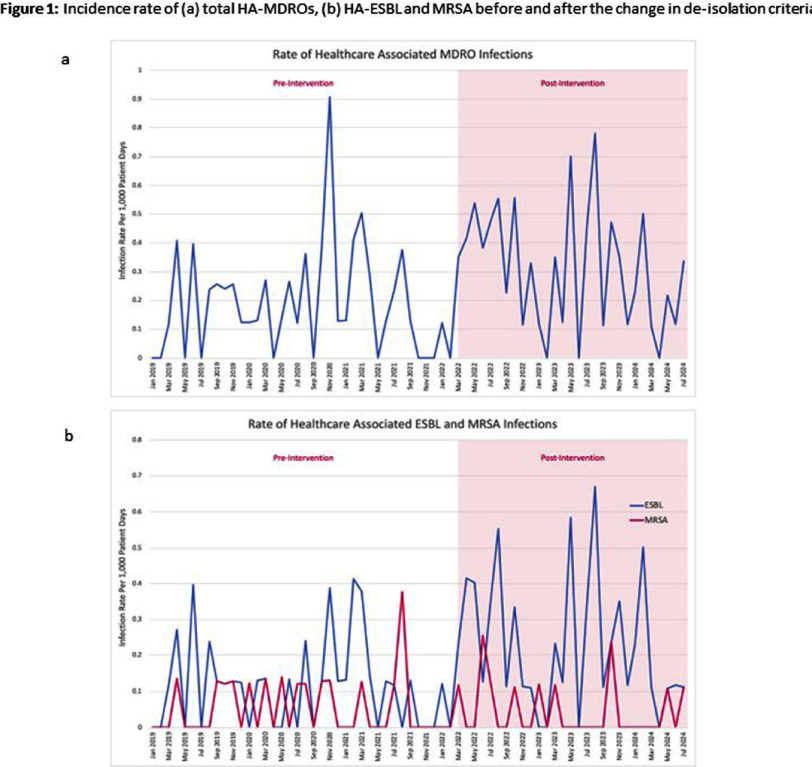

**Figure:**